# Endogenous rhythm and pattern-generating circuit interactions in cockroach motor centres

**DOI:** 10.1242/bio.018705

**Published:** 2016-07-15

**Authors:** Izhak David, Philip Holmes, Amir Ayali

**Affiliations:** 1 Department of Zoology, Tel Aviv University, Tel Aviv 6997801, Israel; 2 Department of Mechanical and Aerospace Engineering, Program in Applied and Computational Mathematics, Princeton Neuroscience Institute, Princeton University, Princeton, NJ 08544, USA; 3 Sagol School of Neuroscience, Tel Aviv University, Tel Aviv 6997801, Israel

**Keywords:** Locomotion control, Central pattern generator, Cockroach, Extracellular-recording, Connectivity model

## Abstract

Cockroaches are rapid and stable runners whose gaits emerge from the intricate, and not fully resolved, interplay between endogenous oscillatory pattern-generating networks and sensory feedback that shapes their rhythmic output. Here we studied the endogenous motor output of a brainless, deafferented preparation. We monitored the pilocarpine-induced rhythmic activity of levator and depressor motor neurons in the mesothoracic and metathoracic segments in order to reveal the oscillatory networks’ architecture and interactions. Data analyses included phase relations, latencies between and overlaps of rhythmic bursts, spike frequencies, and the dependence of these parameters on cycle frequency. We found that, overall, ipsilateral connections are stronger than contralateral ones. Our findings revealed asymmetries in connectivity among the different ganglia, in which meta-to-mesothoracic ascending coupling is stronger than meso-to-metathoracic descending coupling. Within-ganglion coupling between the metathoracic hemiganglia is stronger than that in the mesothoracic ganglion. We also report differences in the role and mode of operation of homologue network units (manifested by levator and depressor nerve activity). Many observed characteristics are similar to those exhibited by intact animals, suggesting a dominant role for feedforward control in cockroach locomotion. Based on these data we posit a connectivity scheme among components of the locomotion pattern generating system.

## Abstract (precis)

**Summary:** Detailed analysis of fictive motor patterns unveils endogenous characteristics of the cockroach thoracic locomotion control networks and their interrelations and enables an explanatory connectivity model.

## INTRODUCTION

Hexapodal locomotion provides insects with stability and flexibility (Ting et al., 1994; Koditschek et al., 2004; Holmes et al., 2006). Like other locomotion patterns, insect gaits emerge from complex interactions among neural activation, muscle and body mechanics, and the environment (Dickinson et al., 2000). Here we studied the relative extent to which these interacting factors are responsible for creating an adaptive functional gait and provide a basis for a comparative investigation of the common nature of the neural architecture shared by other insects.

There is an inherent difficulty in obtaining a functional motor output from isolated central motor circuits. One approach has been to utilize mechanical or chemical stimuli to generate rhythmic activity in the walking-related, thoracic central-pattern-generator networks (CPGs). While the motor output recorded from such preparations is not identical to intact walking, it can still provide useful insights into connectivity patterns, interactions among sub-units and the role of sensory and descending inputs in motor control. This methodology is facilitated by employing a comparative approach and by combining experiments and theory (Ferrell, 1995; Ayali et al., 2015b).

Pearson and Iles (1970) was among the earliest of these studies; by eliciting stepping-like rhythms in a deafferented metathoracic ganglion of *Periplaneta americana* (i.e. deprived of leg-sensory feedback), they provided evidence for the independence of the rhythm from proprioceptive feedback. This and further work by those authors postulated a mechanism of central coupling (mutual inhibition) between burst-generating circuits underlying the rhythmic output (Pearson, 1972; Pearson and Iles, 1973). In those early studies, walking-like rhythms in deafferented preparations were induced by mechanical stimulation (Pearson and Iles, 1970, 1973) or recorded when emerging spontaneously [in only ∼50% of preparations, Pearson and Iles (1973)]. The rhythm was short-lasting, however, and when induced by mechanical stimuli it could have represented reflexive responses rather than spontaneous walking. Following studies of crustaceans (Elson and Selverston, 1992; Chrachri and Clarac, 1990), Ryckebusch and Laurent (1993) used the muscarinic agonist pilocarpine to induce fictive-stepping activity in the locust-isolated thoracic ganglion more reliably and consistently. Independent rhythmic activity in a single hemiganglion, as well as contralateral coordinated activity, suggested CPG-induced leg motor patterns and endogenous coupling in their preparation.

Studies indicated that in stick insects thoracic CPGs are incapable of producing coordinated walking-like motor patterns in the absence of proprioceptive feedback (Bässler and Wegner, 1983; Büschges et al., 1995), supporting the concept that coordination is mainly sensory-dependent (Cruse, 1990; Büschges et al., 2008; Büschges and Gruhn, 2007). Recordings of pilocarpine-induced activity in deafferented ganglia revealed that each hemiganglion comprised at least three discrete CPGs each controlling one of the three main leg-joints, and that these CPGs are at best only weakly coupled with each other or with neighbouring leg CPGs (Bässler and Büschges, 1998; Büschges et al., 1995).

Pilocarpine was also used to induce rhythmic motor patterns by Fuchs et al. (2011); they verified that, in cockroaches, a coordinated motor pattern can be elicited in the absence of sensory feedback (Ayali et al., 2015a). The pharmacologically-induced pattern comprised alternating activity in antagonistic coxal depressor and levator motor neuron (MN) groups, with intersegmental phase relations that share similarities with functional gaits of the intact cockroach.

The above common principles, as well as inconsistencies, were highlighted by various modelling efforts based on the experimental findings from the locust (Ryckebusch et al., 1994; Ryckebusch and Laurent, 1994), stick insect (Cruse et al., 1998; Daun-Gruhn and Tóth, 2011; Tóth et al., 2015) and cockroach (Holmes et al., 2006; Fuchs et al., 2011, 2012; Szczecinski et al., 2014). The theoretical work also revealed some gaps in our understanding of the details of insect locomotion control (e.g. Ayali et al., 2015b). One such gap clearly arises from the lack of a detailed description of the endogenous rhythmic motor pattern of the isolated cockroach thoracic pattern-generating circuits. Such a description is crucial for a complete understanding of the relative role of central circuits, sensory feedback and descending control.

Here we used simultaneous extracellular recordings of levator motor neurons (LevMNs) and depressor motor neurons (DepMNs) (innervating the levator- and depressor-muscles of the coxo-trochanteral joint) from a deafferented nerve cord of *P.* *americana*. Due to technical limitations, and also for comparison with previous studies (cited above) which had tended to focus on the insect meta- and mesothoracic ganglia, we limited the current study mainly to these two ganglia. We describe intrinsic phase relations, coupling strength and its directionality, in nine pairs of homologues and heterologous inter- and intra-hemiganglia pairs of levator and depressor MNs. We also describe the latencies between activity bursts and their overlap, spike frequency of the studied MNs, the levator/depressor ratio, burst durations and duty cycle, as well as the relations of these parameters to the cycle frequency. Such quantitative knowledge is essential in order to decipher the connectivity within and among CPGs and to understand how various inputs shape the endogenous rhythm. This further allows us to re-examine previous suggestions that were based on a more qualitative approach, and to compare our findings to data from intact insects. Finally, we propose a scheme of connectivity for the levator-depressor control networks. The proposed model seeks to bridge between ‘minimal’ models, like that of Pearson and Iles (1973) or Couzin-Fuchs et al. (2015b), and more detailed models, such as those developed for stick insect locomotion (see details in Ayali et al., 2015b and references within).

## RESULTS

### Temporal characteristics of the pilocarpine-induced motor pattern

**Fig. 1.**
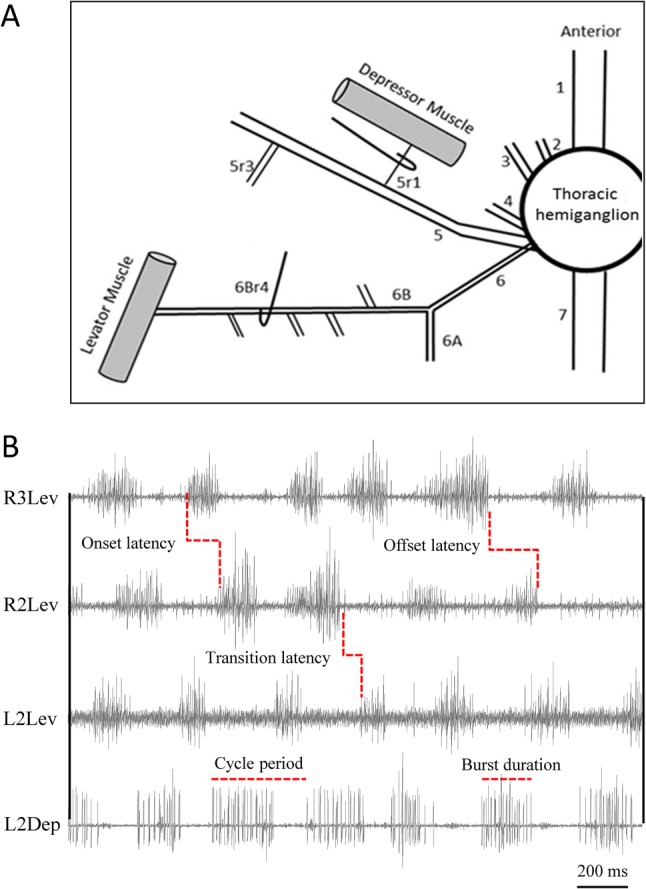
**Electrode positioning and the recorded rhythmic activity.** (A) Schematic presentation of a thoracic hemiganglion and its peripheral nerves. Hooks mark recording sites. Recorded nerves were crushed distal to the recording site in order to prevent the transmission of afferent signals from leg-sensors into the hemiganglionic neuropile. (B) Recording of rhythmic activity of levator and depressor MNs. Nomenclature is presented according to: body side, thoracic segment, and nerve function, e.g. L3Dep, left-metathoracic-depressor. L2Lev and R3Lev burst approximately in-phase with each other, as do R2Lev and L2Dep. Latencies are defined in the text (see ‘Results’). The antagonistic L2Lev and L2Dep alternate in approximate anti-phase, as does the ipsilateral levator pair R2Lev and R3Lev. Average burst duration is greater for depressor in comparison to its antagonistic levator (e.g. L2Dep duration>L2Lev duration).

Our findings regarding the general characteristics of extracellular recordings from the coxa-trochanter levator and depressor nerves (6Br4, and 5r1 respectively; Fig. 1A) were largely in agreement with previous reports (Pearson and Iles, 1970, 1973). Firing patterns were inconsistent, including approximately-periodic bursts of action potentials, but also tonic firing and quiescent periods. For analyses, we selected episodes of approximately periodic bursts in at least two hemiganglia, lasting at least five cycles (mesothoracic or metathoracic, left or right; e.g. Fig. 1B). A total of 205 rhythmic bouts of up to 30 cycles each, in 22 different animals, were analysed (10.43±4.49 cycles per rhythmic bout; hereafter results are presented as mean±s.d. unless noted otherwise). Cycle period (Fig. 1B) was relatively constant within each analysed bout, but varied within and among different animals (80-2245 ms; mean for 205 coefficients of variance=25.63±12.31 ms).

**Fig. 2.**
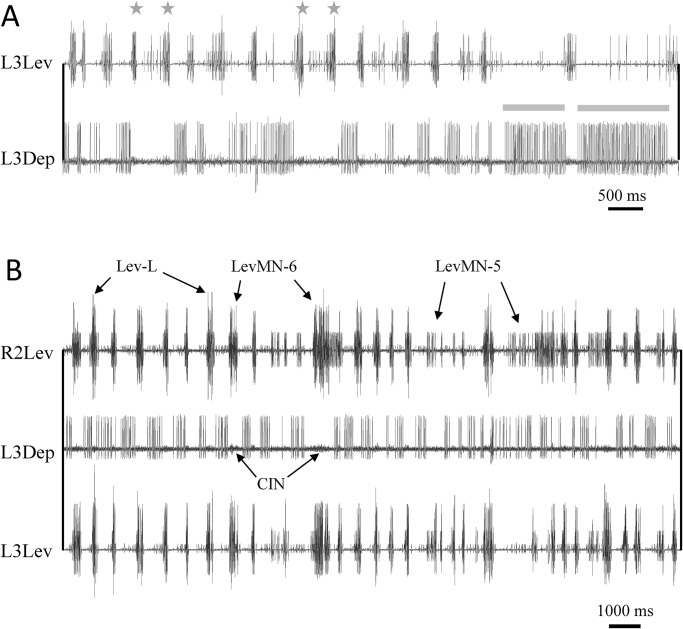
**The pilocarpine-induced motor pattern.** (A) Depressor MNs burst termination is dependent on levator activity. Asterisks above levator trace denote double-bursts; grey bars: depressor tonic firing in the absence of levator bursts. After levator activity halts, the depressor continues to fire tonically for a highly variable time period (seconds-minutes), with highly variable spike-frequency. In addition, multiple bursting was relatively common in levators and rare in depressors. (B) An example of prolonged pilocarpine-induced rhythmic activity in levator and depressor MNs. Activity of levator MNs 5, 6 and larger units (LevMN-5, LevMN-6 and Lev-L, respectively) is marked. The L3Dep trace comprises bursts of DepMN-Ds. The low-amplitude activity seen between depressor bursts is in--phase with levator bursts and represents the activity of common-inhibitory-neurons (CIN). The two diagonal levators burst in-phase with each other and in anti-phase with the left-metathoracic-depressor, in accordance with predicted activity during the double-tripod gait.

In general, rhythmic bursting in levator nerve recordings was more dominant than depressor nerve activity. This was manifested in double- or even multiple-bursts within one cycle (Fig. 2A), or in some cases, levator rhythmic bursts in one nerve with no rhythmic activity in other levator or depressor nerves. Neither patterns were observed in depressor nerve activity which always accompanied bursts in an antagonistic or neighbouring levator, and in the absence of such nerve activity tended to show tonic firing (Fig. 2A), and ultimately a quiescent period that lasted until a levator resumed firing.

Action potentials of LevMNs and DepMNs were threshold-detected (see Materials and methods) to include the activity of LevMNs-5-12 or slow and fast DepMNs [Ds and Df; Nomenclature is based on the units' amplitude, following Pearson et al. (1970); and Pearson and Iles (1970)]. As seen in Fig. 2B, levator bursts always comprised spike trains of LevMN-5, and less consistently of LevMN-6, firing within the burst duration of LevMN-5. Larger amplitude levator units were seldom observed. Depressor bursts mostly comprised DepMN-Ds activity while DepMN-Df was rarely recruited and then only for a few rapid spikes.

**Fig. 3.**
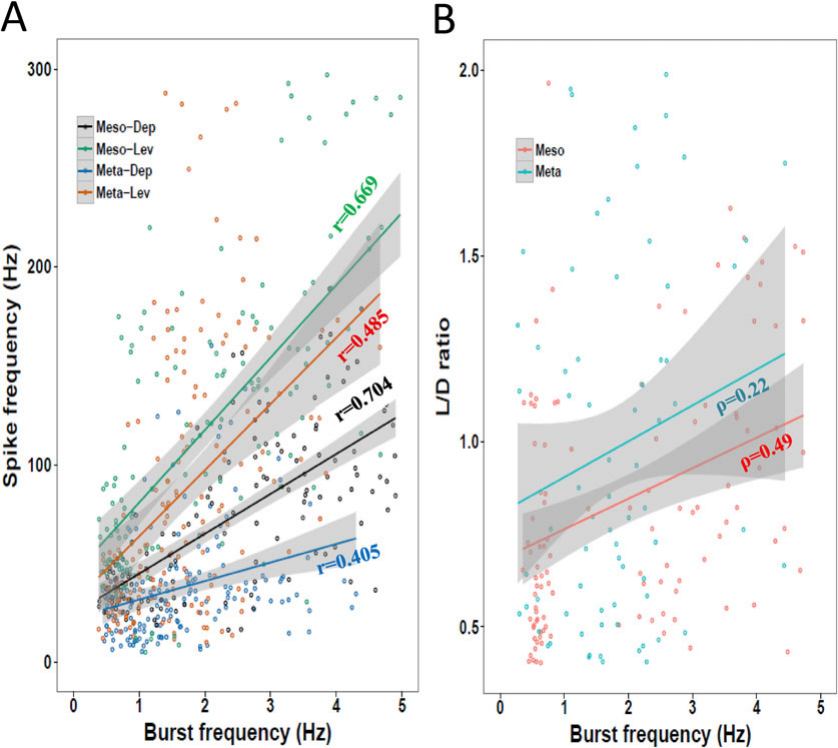
**Endogenous temporal characteristics are dependent on burst frequency.** Grey bands represent confidence interval of the linear regression lines. Correlation coefficients' [Pearson's r (A) and Spearman's ρ (B)] are noted. Data are averaged from left and right hemiganglia. Details of correlations are presented in ‘Results’. (A) Spike frequency is dependent on burst frequency in the absence of proprioceptive or descending inputs. Spike frequency during a burst of activity increases with increasing burst frequency; this dependency is greater for levators than for depressors. Levators of both ganglia exhibit similar spike frequencies and rate of change (slope). Spike frequency significantly differs between the two depressors. (B) L/D ratio positively correlates with burst frequency. The range of recorded frequencies is smaller in the metathoracic samples. L/D ratio is not significantly different between the two ganglia, and in most cases it is greater in the metathorax. The slopes are similar, although the correlation is stronger in the mesothorax.

We measured intra-burst spike frequency (hereafter, spike frequency) comparatively during rhythmic activity of antagonistic MNs in the meso- and metathoracic ganglia. DepMNs spike frequency was lower than that of their antagonistic LevMNs and less variable (levators: 125±113.89 Hz; depressors: 56.91±39.16 Hz; Wilcoxon signed-rank test, *P*<0.001). Mesothoracic MNs showed greater average spike frequency than their metathoracic homologues (Fig. 3A, Table S1; Mann–Whitney test, *P*<0.05). In both LevMNs and DepMNs, spike frequency positively correlated with overall burst frequency, most strongly in the mesothorax (Fig. 3A; Fisher r-to-z transformation, *P*<0.01). The regression slope was higher for LevMNs than for DepMNs and greater for the mesothoracic than metathoracic depressor.

We next examined burst durations, which are of major importance when comparing with the intact motor pattern (where they directly relate to muscle activity), and found that DepMN-Ds average burst durations were longer than those of their antagonistic LevMNs (Lev=312.53±282.84 ms, Dep=344.49±329.72 ms, Mann–Whitney test, *P*=0.031, Fig. 1B). Burst durations of simultaneously active MN pairs (depressors or levators in different hemiganglia) were positively correlated, and more strongly so between MNs active in-phase than those active in anti-phase (mean of correlation coefficients was 0.712 for in-phase and 0.478 for anti-phase; Mann–Whitney test, *P*<0.001). However, since burst durations were positively correlated with cycle period, we focussed on such correlations rather than on the burst durations themselves. The mean correlation coefficient was greater for DepMNs than for LevMNs (DepMNs=0.806, *n*=1013 bursts; LevMNs=0.630, *n*=1288 bursts, Mann–Whitney test, *P*<0.001). Between the different nerves, the correlation was stronger when calculated for DepMNs burst durations and LevMNs cycle periods. (Lev_duration_-Lev_cycle period_: r=0.360, Lev_duration_-Dep_cycle period_: r=0.700, Dep_duration_-Lev_cycle period_: r=0.815).

To further determine the characteristics of the rhythm we compared the duty cycle (burst duration/cycle period) of LevMNs and DepMNs rhythms. We found differences between the two ganglia: unlike the metathorax, the mesothoracic depressor's duty cycle was longer and more variable than that of its antagonistic levator (Table S1, paired *t*-test, *P*<0.005). Moreover, whereas both the mesothoracic and metathoracic levators shared similar duty cycles, those of the mesothoracic depressors exceeded their metathoracic homologues (Student's *t*-test, *P*<0.001).

A commonly used parameter to describe locomotion gait is the ratio between the durations of leg protraction and retraction (P/R ratio). Here we calculated the ratio between antagonistic LevMNs and DepMNs burst durations (hereafter L/D ratio) in order to compare the two ganglia (Fig. 3B), and to compare these to the P/R ratio measured from the intact walking cockroach. The L/D ratio tended to be lower in the meso- than in the metathoracic ganglion (meso=0.820±0.410, meta=0.906±0.550, Mann–Whitney test, *P*=0.068). In both thoracic ganglia the L/D ratio was positively correlated with burst frequency. The slope of the regression line was similar in both ganglia, although the strength of correlation differed between them (Spearman's rank correlation coefficient ρ=0.490 and 0.220 for the mesothorax and metathorax, respectively, *P*=0.007).

### Transitions and latencies between motor units: symmetries and asymmetries

**Fig. 4.**
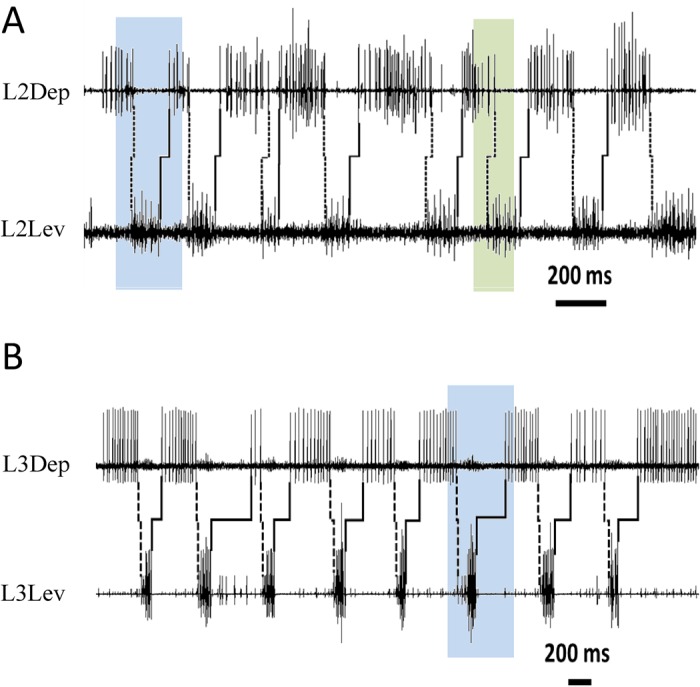
**Transition latency between levator-to-depressor is greater than between depressor-to-levator in antagonist mesothoracic and metathoracic pairs.** Latencies of transition between levator-to-depressor (solid lines) and depressor-to-levator (dashed lines) are asymmetric. Blue: both transitions are shorter in the mesothorax (A) than in the metathorax (B). Green: in the mesothorax, overlap (i.e. negative latency) is much more common in the depressor-to-levator transition than vice versa.

**Fig. 5.**
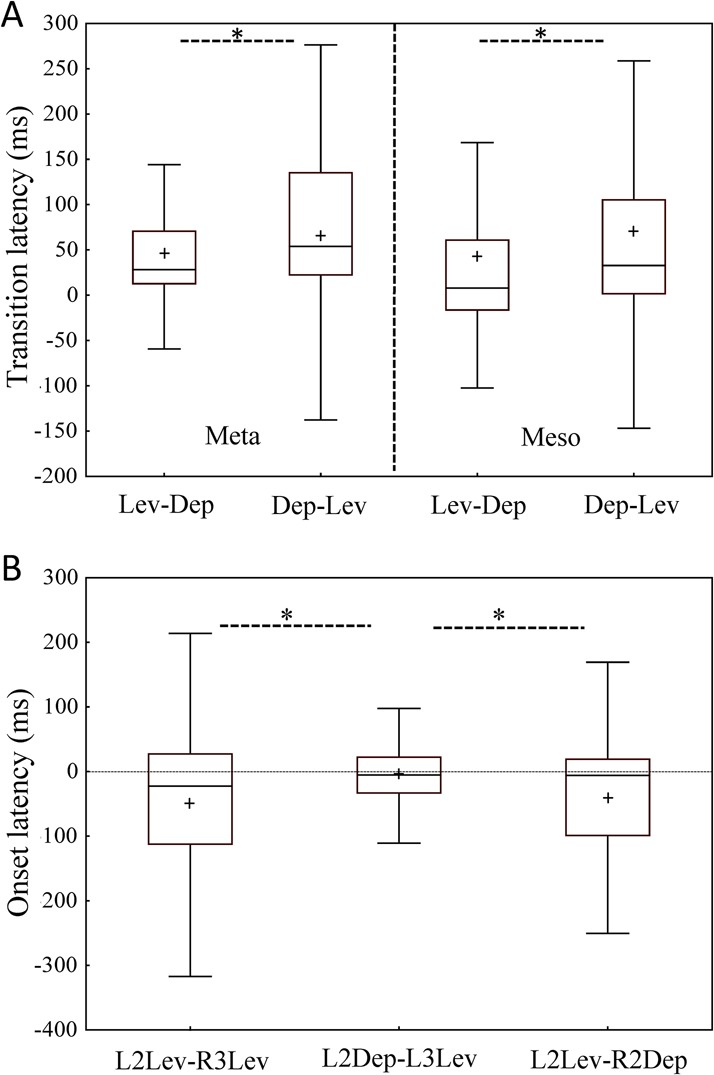
**Latencies within hemiganglia and between neighbouring in-phase MNs.** Line, box and whiskers represent median, interquartile range and non-outlier range (1.5×interquartile range), respectively; means are marked by +; meta, metathoracic; meso, mesothoracic; *P<0.05. (A) Levator-to-depressor transitions are greater than those between depressor and levator in both hemiganglia. Transition latency between Lev-Dep is similar in both hemiganglia, as is the latency between Dep-Lev. However, the significant difference in mean latency between the two different transitions within each pair Wilcoxon signed-rank test might indicate that different mechanisms negotiate each transition. (B) Dashed line: latency=0. From left to right: latencies between burst onsets of diagonal, ipsilateral, and contralateral in-phase active pairs. The ipsilateral pathway exhibits short latency and low variability, indicating strong ipsilateral connectivity. Onset latencies of contralateral and diagonal pairs are similar (*P* > 0.1, Mann–Whitney test). Mesothoracic MN activities precede their metathoracic agonistic MNs, as indicated by the negative means, demonstrating the front-to-back activation sequence that characterizes the double-tripod gait.

To gain further insight into the components of the pattern-generating networks and their interrelations, we next looked at two types of latencies between bursts of activity of antagonistic MNs: the onset latency, which is the latency between the onset of bursts; and the transition latency, which in a walking insect would manifest the transition between the swing and stance phases (the parameters are illustrated in Fig. 1B). We first examined the onset latencies of the antagonistic LevMN-5 and DepMN-Ds (Table S2). In both the examined ganglia, levator-to-depressor onset latencies were greater than depressor-to-levator onset latencies (Wilcoxon signed-rank test, *P*<0.017). In the mesothorax only, the variance of onset latency was greater for Dep-Lev than for Lev-Dep, probably reflecting the more prolonged depressor bursts. When studying the transition latencies, we found that the Lev-Dep latency (end of Lev to start of Dep) was greater than the Dep-Lev latency in both ganglia (Figs 4, 5A, and Table S2; Mann–Whitney test, *P*<0.03). In the metathorax only, transition latency was more variable for Lev-Dep than for Dep-Lev (Levene's test, *P*<0.05). Overlaps between bursts of antagonistic pairs were relatively short (Table S2). The percentage of events in which LevMNs bursts overlapped DepMNs bursts was similar in both ganglia; however, in contrast, DepMNs overlapped their antagonistic LevMNs more frequently in the mesothorax, in comparison to the metathorax (*P*<0.05).

In order to understand better inter-hemiganglia connectivity, burst latencies were similarly examined among neighbouring hemiganglia. We analysed burst onset latencies for ipsilateral, contralateral and diagonal pairs of in-phase active MNs (Table S2). As shown in Fig. 5B, onset latency of the ipsilateral pair approximated the ideal 0 latency, and was significantly shorter and less variable than those of the contralateral and diagonal pairs (Mann–Whitney test, *P*<0.025; Levene's test, *P*<0.05), which differed in their onset latency variances (Levene's test, *P*=0.043). The examined pairs also exhibited differences in the magnitude of positive correlations between burst durations within the paired MNs (the respective correlation coefficients for ipsilateral, contralateral and diagonal pairs are 0.925, 0.664 and 0.810; Fisher r-to-z transformation, *P*<0.001).

Last, we examined the burst transition latencies between neighbouring ipsilateral, contralateral and two diagonal anti-phase pairs (L2Lev:L3Lev, R2Lev:L2Lev, L2Lev:R3Dep, and L2Dep:R3Lev, respectively; Table S2). As expected, the contralateral pair approximated a bilateral symmetry of transition latencies and their variance (Wilcoxon signed-rank test, *P*=0.185; Levene's test, *P*=0.28). The three other pairs exhibited asymmetric transition latencies, which were greater and more variable in the meso-meta direction (i.e. descending) than vice versa (i.e. ascending, Wilcoxon signed-rank test, *P*<0.05). Negative transition latencies or overlaps between bursts were found to be similar in duration in the ascending and descending pathways in three out of four pairs, and somewhat greater in the L2Dep-R3Lev descending pathway (Table S2, Mann–Whitney test, *P*=0.065). Metathoracic bursts overlapped the following mesothoracic bursts more frequently than the other way around; perhaps as a result of the shorter latency in this direction.

### Phase relations and coupling strength

**Fig. 6.**
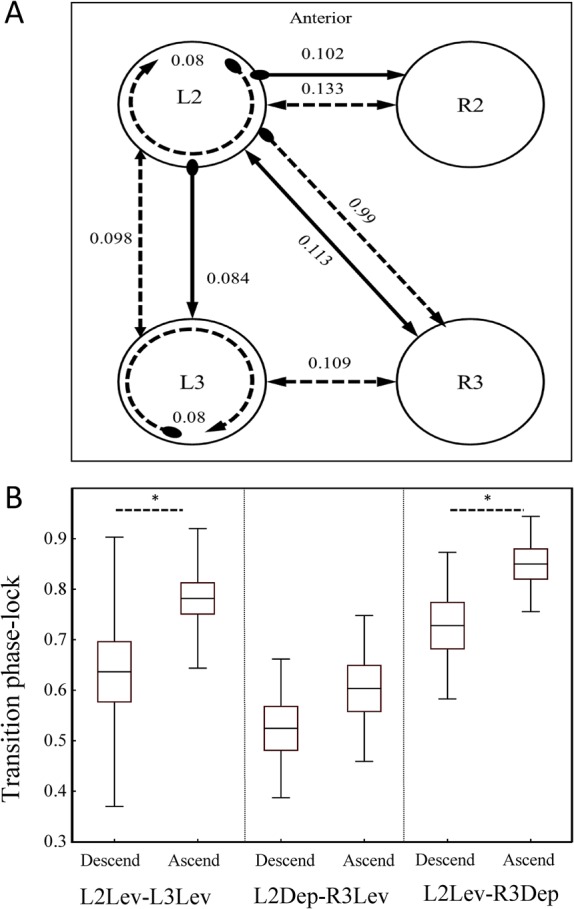
**Phase relations and phase-locking.** (A) Scheme of phase relations s.d., as a measure of coupling strength. Arrow end, levator; round end, depressor; circular connection, coupling between antagonistic MNs within a single hemiganglion. Solid and dashed lines represent pairs of in-phase and anti-phase MN pairs, respectively. Lower s.d. indicates stronger coupling. Endogenous coupling strength was found to be dependent upon these parameters: (i) direction, ipsilateral coupling is stronger than contralateral and diagonal coupling; (ii) hemiganglia involved, contralateral coupling differs between ganglia; and (iii) function of the coupled MNs, coupling between levator and depressor is stronger than between two levators. (B) Phase-locking strength between mesothoracic and metathoracic MNs is asymmetrical and stronger in the ascending pathway. Data for three pairs are normally distributed (Shapiro-Wilk test, *P* > 0.05) and are presented. Line, box, and whiskers represent mean, s.e.m. and s.d., respectively. Significance level is marked as *P<0.05. Transition phase-lock is stronger in the ascending pathway for each of the pairs. Asymmetric phase-locking indicates differences in the mechanisms of coordination in different directions.

Phase relations among muscles or MN activities are commonly used in pattern-generation studies to aid in deciphering the circuit's architecture from its motor output. Here we used the standard deviations of phase differences between MN bursts recorded from different nerves and hemiganglia as manifestations of the strength of coupling between the different underlying oscillatory networks. Our estimation was based on the assumption that strong coupling results in low variability in the phase relations between the oscillators, and vice versa (Boothe et al., 2013; Greene and Spirito, 1979; Rillich et al., 2013). For every pair of MNs, five bouts were sampled from each of five different preparations (*N*=5 animals, *n*=25 bouts, except for L2Dep:R3Lev: *N*=3, *n*=15). To obtain the variability between the selected pairs of MNs, we first calculated the centre-of-gravity (CoG, defined in Materials and methods) of the MN bursts and then the standard deviation of the phase difference between CoGs, separately for each recording bout. Fig. 6A summarizes these standard deviations, and Table S4 summarizes the significance tests among the different groups.

We first examined phase relations and found a left-right symmetry in coupling strength, allowing the pooling of data (e.g. mean phase and variability of R2Lev:R3Lev and L2Lev:L3Lev did not differ, nor did those of L2Lev:R2Dep and R2Lev:L2Dep). Coupling between antagonist pairs within a hemiganglion was found to be similar in the meso- and metathorax and significantly stronger than coupling between different hemiganglia (Mann–Whitney test, *P*<0.05) for all but L2Dep:L3Lev. Inter-hemiganglia coupling of Lev-Dep pairs was found to be greater than that of Lev-Lev pairs. In a comparison between pairs of similar function in different hemiganglia, ipsilateral coupling was found to be stronger than contralateral and diagonal coupling (Mann–Whitney test, *P*<0.05). Moreover, metathoracic coupling was found to be stronger than mesothoracic coupling (Mann–Whitney test, *P*=0.044). We found no significant differences between contralateral and diagonal coupling.

We then further analysed the data to determine whether the coupling is symmetrical, and whether it is reflected differently by burst onset, offset and transition latencies. We used the vector strength (VS) as a measure of the strength of phase-locking, following Boothe et al. (2013). VS ranges between 0 and 1, with 1 corresponding to perfect phase-locking and 0 corresponding to random relations between events. For in-phase pairs, VS was calculated separately based on onset and offset latencies, and the two were compared. No differences were found, indicating that burst onsets and terminations are similarly coupled between MNs that are active in-phase (Table S3, paired *t*-test, *P*>0.1). For anti-phase pairs, VS was calculated separately based on onset and transition latencies (i.e. onset phase-locking and transition phase-locking respectively) to allow comparisons between the various measures. In general, transition phase-locking was greater than onset phase-locking when calculated for the same set of bursts (Table S2).

For all heterogeneous pairs, Lev-Dep onset phase-locking was greater in comparison to Dep-Lev (paired *t*-test, *P*<0.1). Transition phase-locking for antagonistic pairs was similar for Lev-Dep and Dep-Lev (paired *t*-test, *P*>0.3) and also similar in a comparison between the two ganglia. In contrast to this symmetry, the transition phase-locking for the three anti-phase pairs from different ganglia was found to be stronger for the ascending pathway than the descending pathway (Fig. 6B; paired *t*-test, *P*<0.1).

### Phase relations and burst frequency

In order to characterize the endogenous coordination and compare it to that seen during cockroach double-tripod locomotion, we calculated the mean phase relations and their possible correlation with burst frequency (Table S4). Phase relations between antagonistic MNs were very close to the ideal value of Φ=0.5. In-phase pairs burst almost simultaneously, although for the pairs L2Dep:L3Lev and L2Lev:R3Lev the mesothoracic MNs burst slightly before the metathoracic MNs (as also shown in Fig. 5B). Phase relations and burst frequencies were positively correlated and stronger for Lev-Lev pairs than for Lev-Dep pairs (Fisher r-to-z transformation, *P*<0.05). Interestingly, the mesothoracic antagonistic pair exhibited a stronger correlation than the metathoracic pair (Fisher r-to-z transformation, *P*<0.05).

### Connectivity model

**Fig. 7.**
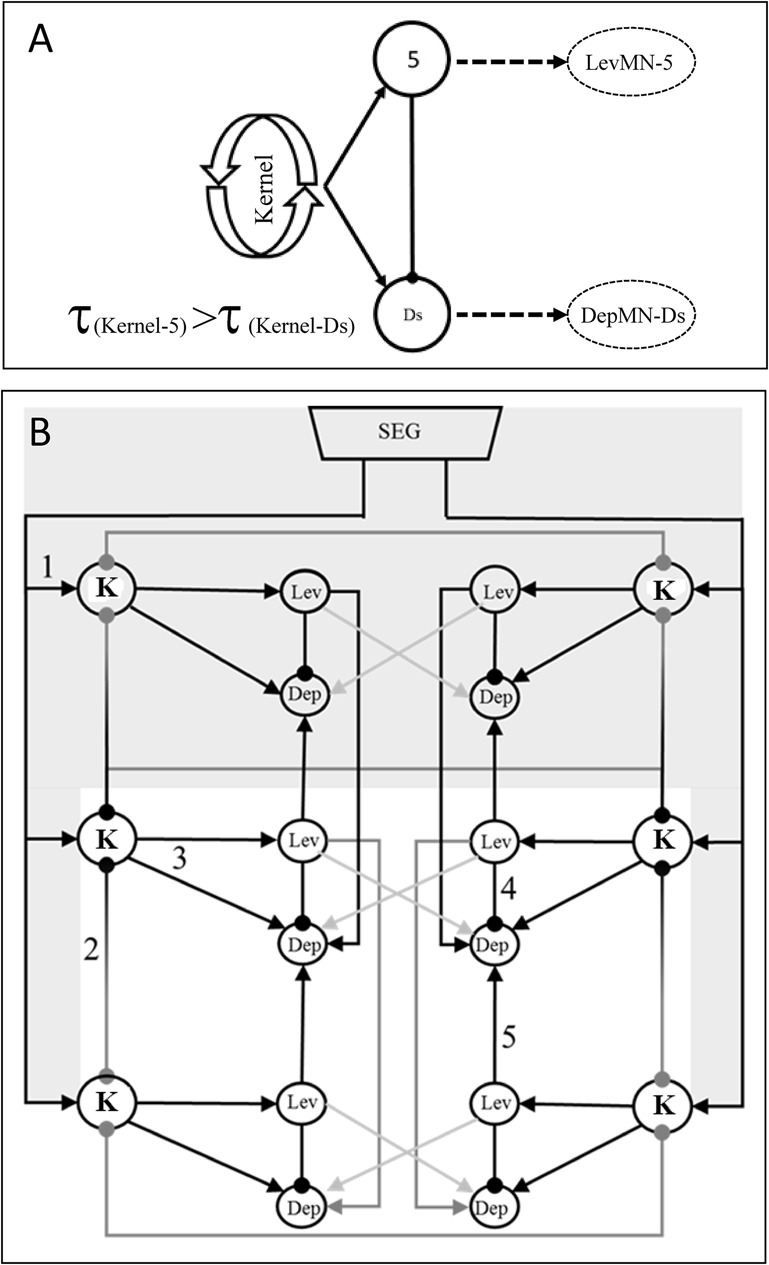
**Schemes of the connectivity within and between the coxa-trochanter CPGs.** (A) A three-component local hemiganglionic control architecture. Arrow end, excitatory synapse; round end, inhibitory synapse; τ, time constant; 5, LevIN-5; Ds, LevIN-Ds; Kernel, oscillating CPG kernel that excites LevIN-5 and DepIN-Ds. LevIN-5 is first to burst and activate its following MN, while inhibiting DepIN-Ds. Due to the suggested greater τ in the kernel-DepIN-Ds path, once LevIN-5 excitation by the oscillating kernel is terminated, DepIN-Ds escapes its inhibition but still receives excitation from the kernel, resulting in a DepIN-Ds burst that induces its follower MN to fire its plateau potentials. (B) A suggested minimal connectivity model of the CPG network generating the double-tripod gait activity pattern of the coxa-trochanter-joints. K, oscillating CPG kernel. Shaded grey: data obtained or postulated from previous research. The model employs nearest-neighbour architecture with a front-to-back propagation sequence that can generate the motor patterns observed in this work, corresponding to straight walking on a smooth horizontal surface. Round and arrow ends represent inhibitory and excitatory synapses, respectively. Grey and black lines represent weak and strong connections [e.g. the direct inhibitory K-K connection descending from the mesothoracic hemiganglia represents weaker (grey) meso-meta connection in comparison to the ascending, stronger (black), meta-meso pathway]. Lev and Dep represent LevIN-5, and DepIN-Ds, respectively. 1, tonic drive generated by the subesophageal ganglion to activate local oscillatory-kernels; 2, mutual inhibition between neighbouring oscillatory-kernels; 3, oscillatory-kernel simultaneously excites LevIN-5, which is first to burst, and DepIN-Ds; 4, LevIN-5 activity inhibits its antagonistic DepIN-Ds; 5, LevIN-5 excites all DepIN-Ds of its neighbouring hemiganglia. The architecture is minimal and allows the addition of various centrally-generated inputs, as well as head-descending and proprioceptive inputs.

Finally, the results of the current work, combined with the relevant literature, enable us to suggest a schematic model of connectivity within an individual leg's coxa-trochanter CPG (Fig. 7A), and between the CPGs of the different legs (Fig. 7B). This model seeks to account for the double-tripod-like patterns resembling unperturbed ‘normal walking’ [straight-walking on a smooth horizontal surface; (Delcomyn, 1985)]. Its minimal architecture enables the addition of the various inputs, centrally- or sensory-generated, that are required in order to explain the variety in the cockroach's locomotion-related behaviours.

### The local leg alternating unit

Since the antagonistic LevMNs and DepMNs are not directly connected (Pearson and Iles, 1970), their activity must be coordinated through pools of driving interneurons (INs). For simplicity, these levator and depressor IN pools are represented here as single INs. Hence, the basic unit that our model considers is the antagonistic pair of individual leg levator and depressor INs (i.e. LevIN and DepIN, respectively), innervating their corresponding MNs. This suggested basic unit is consistent with our findings that: (i) the strongest coupling is between the intra-hemiganglion antagonistic pairs; (ii) phase differences of such pairs show almost perfect anti-phase activity; and (iii) negative correlations exist between duty cycles of pairs of antagonistic MNs.

Our findings suggest that LevMN-5 and LevMN-6 are the only bursting LevMNs that alternate with DepMN-Ds. Within the levator bursts, LevMN-5 was first to fire and it maintained its activity throughout the entire burst duration. LevMN-6 never burst during LevMN-5 quiescent periods, consistent with previous reports (Pearson, 1972; Pearson and Iles, 1970). Since our model is based on direct INi-to-MNi innervation (e.g. LevIN-5 innervates LevMN-5), we consider LevIN-5 to be the basic alternating levator unit, while LevIN-6 supports it to sharpen transitions and increase muscle torque (Watson and Ritzmann, 1998b) or to adapt to an increase in load (Pearson, 1972). Depressor activity observed in this work always comprised DepMN-Ds activity, while DepMN-Df firing was less frequent and inconsistent. Hence, our model considers DepIN-Ds to be the basic depressor unit, while Df supports Ds activity at faster running speed (Delcomyn and Usherwood, 1973; Gal and Libersat, 2006; Pearson, 1972; Watson and Ritzmann, 1998b), during startle response (Levi and Camhi, 1996) and immersed locomotion (Gal and Libersat, 2006). We note that the generally low stepping frequencies observed may not require much Df activity.

Our data also show that when LevMN-5 rhythmic bursting ceased, the antagonistic DepMN-Ds frequently continued tonically. Moreover, while the relatively constant burst durations of LevMN-5 were consistent with an oscillating bursting neuron pattern, those of DepMN-Ds were more typical of a tonically-spiking neuron that fires between its inhibition periods, as also seen in intact cockroach depressor EMG recordings (Gal and Libersat, 2006), in the deafferented stick insect (Büschges, 1998) and in locust *in vitro* preparations (Ryckebusch et al., 1994). Hence, we suggest that DepMN-Ds might be plateau neurons, similar to the findings of Hancox and Pitman (1991, 1993) regarding DepMN-Df. Using intracellular recordings from DepMN-Df soma, in the cockroach isolated hind ganglia, those authors demonstrated that DepMN-Df generates plateau potentials in response to direct cell membrane stimuli and to presynaptic stimulation. Activities in these bi-stable neurons can persist beyond the stimuli that evoked them, in either state (i.e. on or off), until a new stimulus arrives (review by Marder, 1991). Thus, in our model, temporally-short DepIN-Ds firing can induce prolonged DepMN-Ds plateau spike trains.

### Generating the rhythm

Locomotion-related rhythmicity usually arises from circuit interactions between neurons that are not themselves rhythmic. The simplest version comprises two reciprocally-inhibitory neurons that generate rhythmic activity (Getting, 1989; Daun et al., 2009), as in the well-studied half-centre oscillator (HCO) model (Marder and Bucher, 2001). The antagonists LevIN-5 and DepIN-Ds could potentially form the two sides of such an HCO; although, if rhythm emerges due to mutual inhibition between them, the rhythmic activity of LevMN-5 should be accompanied by the anti-phase activity of antagonistic DepMN-Ds. However, in many episodes LevMN-5 was rhythmically active while its antagonist depressor remained quiescent, suggesting that direct mutual inhibition between these antagonists is unlikely.

Following the Ghigliazza and Holmes (2004) complete CPG model, which utilizes phase reduction to enable the collapse of more complex networks into a simplified oscillating kernel (Holmes et al., 2006, Kukillaya et al., 2009), and for simplicity, we represent this oscillatory CPG kernel as a single bursting oscillating IN (‘Kernel’; Fig. 7A), which serves as the local proxy of the central control network.

We assume that each of these kernels enables independent activity of the hemiganglionic network. This assumption is supported by our many observations of alternating activity in a single hemiganglion during long quiescent periods in neighbouring hemiganglia, as well as by the rhythmic activity recorded from isolated thoracic ganglia (Pearson and Iles, 1970; Büschges, 1998; Ryckebusch and Laurent, 1993). The presence of a hemiganglionic joint-specific timing mechanism has also been suggested for the stick insect (Büschges, 2005) and for *B. discoidalis* (Szczecinski et al., 2014).

### The hemiganglionic coxa-trochanter control circuit

Previous research in cockroaches and locusts has suggested that levators are centrally controlled (Pearson and Iles, 1970, 1973; Ryckebusch et al., 1994). Our findings in this respect indicate that:

- LevMNs can burst independently of DepMNs or other LevMNs, as previously reported for the cockroach (Pearson and Iles, 1970) and locust (Ryckebusch and Laurent, 1994).
- As in the locust (Ryckebusch and Laurent, 1994), DepMN bursts in the cockroach also occurred only in conjugation with LevMN bursts in at least one hemiganglion. This suggests that the depressor is not timed solely by a central controller. This also corresponds well to Pearson's report that sensory feedback shapes the motor output mostly by affecting the DepMNs output (Pearson, 1972).
- Levator spike frequencies exhibit stronger correlations with burst frequency than depressors, with relatively low variability of burst durations, as also found in the stick insect (Büschges et al., 2008) and locust (Ryckebusch et al., 1994).
- Dep-Lev transition latency is shorter and less variable than that of Lev-Dep. This difference can be explained by a central-controller innervation that onsets LevIN-5 to burst, while DepIN-Ds onsets and offsets are not directly timed by the oscillatory-kernel, thus presenting greater and more variable average latency from the preceding levator burst.
- Unlike depressors, bursting activity of LevMNs in different ganglia exhibited similarities in burst duration, duty cycle and frequency of overlap with antagonistic DepMNs.

These similarities in LevMNs activity can result from either strong coupling between neighbouring LevINs, or from central control over LevINs activities. Our data suggest that neighbouring LevMNs exhibit relatively weak mutual influences; specifically, we found weak correlation between burst durations, weaker Lev-Lev coupling in comparison to Lev-Dep pairs, and weak correlation between LevMN cycle periods and neighbouring LevMN burst durations in contrast to the strong correlation between DepMN-Ds burst duration and LevMN cycle periods. In addition, although neighbouring LevMN duty cycles were similar, they were not correlated. This is in contrast to the negative correlation found within heterogeneous anti-phase pairs.

These findings indicate that the cockroach's neighbouring levators do not directly influence one another, as was also suggested for the stick insect (Dürr et al., 2004). Consequently, we suggest that the similarities between characteristics of neighbouring LevINs are more likely to result from their being under direct control of the local oscillatory-kernel, which in turn is coupled with neighbouring oscillatory-kernels.

Due to the above findings, our local network model was designed to comprise LevIN-5, DepIN-Ds, and an oscillatory-kernel that directly excites its adjacent LevIN. The question remains as to what causes DepIN-Ds, and subsequently DepMN-Ds, firing. A three-component mechanism, termed by Getting (1989) as ‘parallel excitation/inhibition network’, may explain the output we observed here. A similar scheme was suggested for the locust locomotion control network (Ryckebusch et al., 1994). Our suggested architecture includes an oscillator that simultaneously excites DepIN-Ds and LevIN-5, which is first to burst, while simultaneously inhibiting DepIN-Ds. In this case, if the oscillatory-kernel burst duration is longer than that of LevIN-5, then DepIN-Ds will continue to receive the excitatory drive when the levator inhibitory output is dwindling or terminated, resulting in a short DepIN-Ds firing that can drive DepMN-Ds to generate plateau potentials. Our finding of Lev-Dep overlap (>20% of events, Table S2) suggests that this inhibitory current dwindles as the levator oscillation approaches termination. In such an architecture, prolonged oscillator bursts will increase the probability of a levator double-burst, as indeed was frequently observed.

### Mutual inhibition between neighbouring oscillatory-kernels and the role of the subesophageal ganglion

To present a more complete architecture, we have incorporated the current knowledge of subesophageal ganglion (SEG) descending inputs, and our suggested connectivity between oscillatory-kernels, into our model scheme.The SEG is known to play a critical role in initiating and maintaining cockroach walking and enabling proper leg coordination (Gal and Libersat, 2006) by means of tonic-descending inputs that increase the excitability of thoracic ganglia CPGs and participate in walking-speed regulation (Gal and Libersat, 2010a,b; Ritzmann et al., 2005; Ridgel and Ritzmann, 2005; Schaefer and Ritzmann, 2001). These inputs were also found to have an inhibitory effect on stance-phase activated MNs through reflex modulation (Mu and Ritzmann, 2008). The excitatory effect on the swing-phase, including cycle period regulation, was not attributed to reflex mechanisms, thus supporting the notion of central control over the LevINs.

SEG-tonic-descending inputs are assumed here to directly affect all the thoracic oscillatory-kernels equally (Fig. 7B), and the double-tripod pattern is achieved through mutual inhibition between neighbouring oscillatory-kernels. This assumption is supported by findings from locust *in vitro* preparations (Ryckebusch and Laurent, 1994) where, following an insect's thoracic connective amputation, rhythm emerges in previously quiescent hemiganglia and that in previously-active hemiganglia the rhythm becomes more regular while burst duration variability decreases. In contrast to our own findings, Pearson and Iles (1973) suggested a mechanism of mutual inhibition between ipsilateral levators burst-generating networks. However, mutual inhibition between the oscillatory-kernels can resolve this contradiction. Thus, a double-tripod pattern emerges from a front-to-back propagation sequence, in combination with ipsilateral and weaker contralateral mutual inhibition between neighbouring oscillatory-kernels. Due to conduction distances, the resultant in-phase bursting of ipsilateral pro- and meta-thoracic oscillatory-kernels should exhibit phase relations approximating 1, as indeed found in the walking cockroach (Delcomyn, 1971).

### Connectivity between LevINs and DepINs from different hemiganglia

Centrally-generated coordination signals have been identified in cockroach thoracic connectives that were in-phase with ipsilateral LevMN activities (Pearson and Iles, 1973). Severing a thoracic connective has been shown to affect leg coordination in the cockroach (Greene and Spirito, 1979; Hughes, 1957), stick insect (Dean, 1989) and locust (Ryckebusch and Laurent, 1994). Based on our findings and previous work, we posit six basic rules of connectivity between LevINs and DepINs in the different hemiganglia (illustrated in Fig. 7B):

- LevIN-5 excites neighbouring DepINs-Ds. This ensures that when a leg lifts in swing, its neighbouring legs simultaneously perform a power-stroke to support the body weight and maintain balance. Our finding of a strong Lev-Dep coupling, also found in the locust (Ryckebusch and Laurent, 1993, 1994), supports this property. In addition, onset phase-locking in contralateral and ipsilateral heterogeneous pairs was found to be stronger for Lev-Dep than Dep-Lev. Moreover, DepMN-Ds burst only in the presence of neighbouring LevMN-5 activities, which suggests that LevIN-to-DepIN innervation is excitatory. The near zero onset latency between the mesothoracic depressor and its ipsilateral levator supports this rule i and rule ii.
- Ipsilateral connections are stronger than contralateral ones, as an intrinsic feature.
- Based on the stronger LevMN-DepMN coupling, in comparison to that of LevMN-LevMN, our model considers LevIN-DepIN coupling as direct, and LevIN-LevIN coupling as indirect and achieved through mutual inhibition between oscillatory-kernels.
- Metathoracic coupling is stronger than mesothoracic coupling. A weak correlation between burst durations of mesothoracic contralateral MNs, along with our findings shown in Fig. 6A, supports this rule.
- Diagonal coupling is functional and not through direct innervation. This rule derives from the network's nearest-neighbour architecture, as inferred from Greene and Spirito (1979) and in agreement with Couzin-Fuchs et al. (2015b).
- Meta-meso ascending coupling is stronger than meso-meta descending coupling. This rule is supported by our finding of shorter and more variable descending transition latency (as in Pearson and Iles, 1973), and greater ascending coupling.

## DISCUSSION

In this study we sought to characterize the endogenous, pilocarpine-induced, rhythmic activity generated by the deafferented cockroach thoracic pattern-generating circuits. Our findings are expected to fill in gaps in our current knowledge, while re-examining previous findings, establishing general rules for connectivity and finally suggesting a parsimonious model that can explain the observed data without compromising the system's ability to generate locomotion-related behaviours. Moreover, our results enable comparisons with data obtained from other preparations and other insects.

### Deafferented versus non-deafferented preparation-feedforward-control dominancy

The cockroach utilizes the double-tripod motor pattern for practically all modes of locomotion, for stride frequencies above 1 Hz (Hughes, 1952; Delcomyn, 1971; Spirito and Mushrush, 1979; Full and Tu, 1991), even including swimming (Cocatre-Zlgien and Delcomyn, 1990). In double-tripod walking, the ipsilateral front and rear legs move in-phase with the contralateral middle leg to form two tripods that alternate in anti-phase (Wilson, 1966). This gait relies predominantly on feedforward control and the non-linear viscoelastic properties of musculoskeletal structures, requiring little or no sensory feedback in order to maintain the functional phase relations among the network's components (Fuchs et al., 2011; Jindrich and Full, 2002; Kukillaya and Holmes, 2007; Kukillaya et al., 2009; Sponberg and Full, 2008; Couzin-Fuchs et al., 2015a; but see Ayali et al., 2015a). The marked and many similarities between our current results and those obtained from intact or semi-intact cockroach preparations further support the double-tripod gait as the cockroach's default endogenous gait.

Similar to findings from behaving *B. discoidalis* electromyogram recordings (Watson and Ritzmann, 1998a,b), we found a positive correlation between DepMNs spike frequency and burst frequency. This endogenous plasticity serves to increase leg-muscle power to match the increase in walking velocity, with shortening of the depressor duration that accompanies an increase in speed being compensated by a centrally-generated increase in burst intensity. Our results show an even greater plasticity in LevMNs, perhaps to support an increase in stride length during fast running (Ting et al., 1994).

In general, phase relations were found to be consistent with those reported for the walking cockroach (Delcomyn, 1971; Pearson and Iles, 1973; Reingold and Camhi, 1977; Spirito and Mushrush, 1979). As also found in the intact *P. americana* and *B. discoidalis* (Bender et al., 2011; Delcomyn, 1971), our recorded in-phase activity indicated a front-to-back activation sequence, in which MNs burst slightly before their caudal ipsilateral counterparts, resulting in phase relations approximating Φ=1. This leg-activation sequence characterizes the double-tripod gait (Bender et al., 2011; Wosnitza et al., 2012) and contrasts the back-to-front pattern found in other common insect gaits (Borgmann and Büschges, 2015).

In addition, variability of phase differences was used here as a measure of coupling strength. Coupling is known to be modulated by both sensory feedback and the behavioural context (Dürr, 2005; Fuchs et al., 2011, 2012) which are parameters that were not expected to influence the output of our brainless deafferented preparation. As commonly found in walking arthropods (Cruse and Knauth, 1989; Borgmann et al., 2007; Sillar et al., 1987; Müller and Cruse, 1991; Dürr, 2005), and embedded in insect (Daun-Gruhn and Tóth, 2011) and vertebrate (Rybak et al., 2015) locomotion control models, ipsilateral coupling was found here to be stronger than contralateral coupling. Diagonal coupling and contralateral coupling presented similar strength, as in the walking stick insect (Cruse and Knauth, 1989), although they differed in latency variability, overlap duration and correlation between the paired MNs durations. In addition, contralateral coupling was found to be stronger in the metathorax than in the mesothorax, as reported for the intact cockroach (Couzin-Fuchs et al., 2015b) and locust *in vitro* preparation (Ryckebusch and Laurent, 1993), although in the intact cockroach this difference varies among preparations and is presumably speed-dependent. Our finding of intrinsic contralateral coupling, although weak, contradicts previous suggestions that it is purely mechanical (Pearson and Iles, 1973), or totally absent in the mesothorax (Greene and Spirito, 1979).

In contrast to findings reported by Couzin-Fuchs et al. (2015b) from the intact cockroach, our deafferented preparations revealed no significant correlation between coupling strength and burst frequency, suggesting that this correlation is mediated through proprioceptive feedback. However, in agreement with findings reported for the intact cockroach (Hughes, 1952, 1957; Delcomyn, 1971), we found a positive correlation between phase relations and burst frequencies, up to 5 Hz, with phase differences in anti-phase activity increasing towards Φ=0.5. This correlation was stronger in the ipsilateral pair of levators, in accordance with Delcomyn (1971). The speed-dependent transition towards the ideal double-tripod gait appears to be encoded in the intrinsic thoracic locomotion control network of the cockroach.

The greater average burst durations of depressors compared to levators are consistent with reports on walking (Reingold and Camhi, 1977; Delcomyn, 1971) and semi-intact cockroaches (Pearson and Iles, 1970), as is the positive correlation found between burst duration and cycle period which was stronger for depressors than levators (Delcomyn, 1971). Moreover, this strong correlation suggests that the levator cycle period is the best predictor of depressor burst duration. Hence, similar correlations between both swing or stance durations and cycle period can be observed in a deafferented network, suggesting that it is an intrinsic property of the thoracic locomotion control network. The depressors' greater and more variable burst durations can also explain our finding of greater Lev-Dep onset phase-locking in comparison to Dep-Lev.

While we show that the levators from the different ganglia share similar characteristics, the mesothoracic depressor presents greater burst duration and duty cycle than its metathoracic homologue, resulting in a somewhat lower mesothoracic L/D ratio. This finding, as well as the finding that faster endogenous rhythms result in an increased L/D ratio, is similar to the previously reported characteristics of the P/R ratio during faster rhythms in the intact cockroach (Spirito and Mushrush, 1979; Delcomyn, 1971).

An additional similarity to intact preparations worth noting is that the Lev-Dep transition latency in our study was found to be greater and more variable than the Dep-Lev latency, in agreement with a study utilizing EMG recordings of levator and depressor muscles in walking cockroaches (Reingold and Camhi, 1977). This could also indicate hyperpolarizing inputs to DepMN-Ds (or its driving interneurons), which alongside common-inhibitory-neurons (CIN) (Fig. 2B; Pearson and Bergman, 1969; Pearson and Iles, 1971), ensures a fast relaxation of depressor muscles in order to minimize resistance to a following leg lift-off.

In contrast to these multiple similarities, we found that coupling was stronger in the meta-meso direction than in the meso-meta direction, in disagreement with the work by Fuchs et al. (2011) on deafferented preparations. However, the finding by Couzin-Fuchs et al. (2015b) that coupling strength ratios are speed-dependent may account for this discrepancy. In addition, our deafferented pilocarpine-stimulated preparation generated low frequency rhythms in comparison to those measured in the intact cockroach (Couzin-Fuchs et al., 2015a,b; Delcomyn, 1971).

Overall, the above similarities emphasize the instrumental role of feedforward control in cockroach locomotion; while the reported differences can contribute to a more specific understanding of the role of various inputs in shaping the CPGs rhythms into the outputs observed in freely-walking cockroaches.

### Differences between the meso- and metathoracic ganglia

The meso- and metathoracic ganglia can be expected to exhibit differences in control network characteristics due to the different functional roles of the middle and hind legs during locomotion (Watson and Ritzmann, 1998b; Full et al., 1991). While the hind legs' main role is to generate propulsion, the smaller middle legs act mainly as stabilizers or as an axis-leg during turning (Quimby et al., 2006). In the stance phase of each step cycle, the middle leg bears the load of its sagittal half of the body while the other half is borne by two legs - front and hind. Interestingly, we found that mesothoracic DepMN-Ds exhibited greater spike frequencies than their metathoracic homologues, thus generating relatively greater depressor muscle-power to support the body load, consistent with the mesothoracic legs' main roles (Jindrich and Full, 1999; Sponberg et al., 2011a,b). This correlation was stronger in the mesothorax, indicating a critical role of this ganglion in gait modification.

Thoracic CPG outputs are also expected to differ because of differences in their connectivity: mesothoracic hemiganglia receive at least one more thoracic input in comparison to the pro- or metathoracic hemiganglia. While a metathoracic hemiganglion is connected with one ipsilateral (rostral) and one contralateral hemiganglion, a mesothoracic hemiganglion is connected with two ipsilateral (rostral and caudal) and one contralateral hemiganglion. Therefore, in our model the mesothoracic oscillatory-kernels and DepINs receive an extra inhibitory and excitatory input, respectively.

The extra input to the mesothoracic DepIN-Ds can explain some of the differences found between the two ganglia. First, mesothoracic DepMN-Ds exhibited greater burst durations and duty cycles than their metathoracic homologues. Inhibition of mesothoracic DepIN-Ds by antagonistic LevIN-5 is enabled when its excitatory inputs decrease following termination of the excitation it receives from its neighbouring LevINs. The network's front-to-back activation sequence means that for mesothoracic DepIN-Ds, the descending input is terminated before the ascending one, resulting in a delay in its antagonistic LevIN-5's ability to inhibit DepIN-Ds, and subsequently in greater mesothoracic DepIN-Ds burst duration and duty cycle. Functionally, this can serve to ensure that the mesothoracic leg remains in stance while its ipsilateral legs are in swing.

The same mechanism can explain the fourfold increase in the frequency of mesothoracic Dep-Lev overlaps, in comparison with the metathoracic one: mesothoracic LevIN-5 onset should inhibit its antagonistic DepIN-Ds; however, the metathoracic LevIN-5 delayed termination can disrupt the hyperpolarization of the mesothoracic DepIN-Ds, resulting in more frequent mesothoracic Dep-Lev overlaps. In the intact walking cockroach this overlap is avoided (Reingold and Camhi, 1977), probably due to the activity of common-inhibitor-neurons and proprioceptive afferents (Iles and Pearson, 1971; Zill and Moran, 1981; Zill et al., 2009).

The greater number of mesothoracic inputs can also account for the weaker mesothoracic coupling we observed here (i.e. noisier phase relations) and which was previously also reported for locusts (Ryckebusch and Laurent, 1994). Such variability may allow better adaptability, which serves the middle legs during straight locomotion as well as during turnings and recovery from perturbations.

Another difference observed between the ganglia was that the ascending transition latency was shorter and less variable than the descending one. This phenomenon was previously observed in the stick insect (Cruse, 1990) and attributed to sensory feedback mechanisms. This is consistent with the general notion that stick insect coordination is primarily achieved through local sensory feedback mechanisms (Borgmann et al., 2011). In contrast findings from deafferented cockroach and locust *in vitro* preparations (Pearson and Iles, 1973; Ryckebusch and Laurent, 1994), in combination with our current findings, suggest that in these insects this asymmetry is a centrally-generated feature that might serve to decrease the probability that a rear leg will swing before the onset of the ipsilateral middle leg's power stroke. This phenomenon has been explained by an inhibitory coupling mechanism followed by asymmetric delayed excitation between ipsilateral neighbouring legs (Pearson and Iles, 1973; Cruse, 1990). We suggest here an asymmetric delayed inhibition between ipsilateral oscillatory-kernels as an alternative explanation. A greater time-constant in the inhibitory pathway between the meso- to-meta-oscillatory-kernels, in comparison to the opposite ascending direction, can result in a delay in the meta-kernel escape/release from inhibition. Similarly, transition phase-locking was found to be greater in the meta-meso-thorax direction. This is in contrast to onset phase-locking, which was found to be more dependent on MN identities (Lev or Dep) than on the direction.

### Connectivity model

We have further employed our findings from this work to present a connectivity scheme that can explain the recorded output. We have incorporated our experimental data, obtained from meso- and metathoracic MNs, into a more complete scheme, including the SEG and prothoracic ganglia (shaded grey in Fig. 7B) that also draws on results and assumptions from earlier studies. The proposed architecture is expected to provide a useful tool for further discussion of our current findings, as well as in guiding future work, such as developing it into a more detailed mathematical model than that of Ghigliazza and Holmes (2004).

## MATERIALS AND METHODS

Experiments were conducted on 22 adult male *Periplaneta americana* cockroaches obtained from our colony at Tel-Aviv University. Animals were kept in 60-litre plastic cages at a room temperature of 30°C, light:dark cycle of 12 h:12 h. Cockroach diet comprised dry cat food (La-Cat, BioPet, Israel) and water *ad libitum*.

### Neurophysiological procedures

The neurophysiological procedures followed Fuchs et al. (2011) with some adaptations: all legs were amputated between the coxa rim and the thorax. The head capsule was opened and the circumesophageal connectives were severed. Silver wire (0.076 mm) hook electrodes were manipulated to hold the coxa-trochanter levator and depressor nerves [6Br4 and 5r1, respectively; (Pearson and Iles, 1970)], as illustrated in Fig. 1A. The recording site was coated with Vaseline for insulation and a reference electrode was inserted into the abdomen. Deafferentation was achieved by crushing the recorded nerves distal to the recording site, and severing all other peripheral nerves exiting the ganglia. The preparation was then placed in a dorsal-side-up position and allowed to recover for at least 20 min.

Several measures were undertaken to ensure that the recorded activity represented locomotion and not righting, grooming or searching behaviour [see also Ayali et al.(2015a)]. Following the finding in Zill (1986) that righting activity is known to emerge only if the animal is positioned ventral-side up, and studies showing that the cockroach's cerci sense gravity (Walthall and Hartman, 1981; Hartman et al., 1987), we fixed our preparations dorsal-side up with intact abdominal connectives. In addition, since grooming is performed using a single leg, we analysed only recordings of simultaneous rhythmic activity in at least two hemiganglia. Moreover, searching behaviour, in its initial moments, can also be mistaken for walking (Delcomyn, 1987). Consequently, our recording sessions started at least 20 min after the preparation has been set in place, thus eliminating the possibility of mistaking searching for walking.

### Pharmacology

Preparations were injected with 500 µl of the muscarinic agonist pilocarpine (1*10-4 molL-1, pilocarpine-HCl 99%, Sigma Aldrich, Rehovot, Israel), freshly prepared in cockroach saline [saline composition follows Becht et al. (1960)]. Pilocarpine is known to non-specifically activate premotor networks of thoracic MNs in deafferented arthropods' thoracic ganglia (Büschges, 1998; Buhl et al., 2008), and was used to induce reliable long-lasting rhythmic activity in leg-motor neurons of *P. americana* (Fuchs et al., 2011), *Manduca sexta* (Johnston and Levine, 2002), *C. morosus* (Büschges et al., 1995), and *S. americana* (Ryckebusch and Laurent, 1993). Although pilocarpine activates both flight- and walking-CPGs, the two networks do not affect one another's output (Rillich et al., 2013). In the cockroach, pilocarpine was shown to increase walking behaviour without initiating changes in inter-segmental coordination (Ridgel and Ritzmann, 2005).

### Data acquisition and analysis

Recording sessions lasted 1-8 h, depending on the animal's response and vitality. Analog voltage was recorded at 10 kHz using two four-channel differential amplifiers (model 1700, AM systems, Carlsborg, WA, USA), a signal conditioner (cyberamp 380, Axon Instruments, Union City, CA, USA), and a 16 bit A-D converter (Digidata 1322A, Axon Instruments, Union City, CA, USA). The signal was recorded and played back in real time using Axoscope software (Molecular Device, Sunnyvale, CA, USA) and processed using DataView software (W.J. Heitler, University of St. Andrews, Scotland). Data were chosen for analysis based on two criteria: (i) recorded bouts showing simultaneous rhythmic activity [as defined in Pearson and Iles (1970)] in at least two hemiganglia, for at least five cycles; and (ii) instantaneous frequencies calculated between bursts recorded in the two nerves had to be consistent throughout the entire recording bout (variation <25%). Bursts were only included if they comprised four spikes or more, and were terminated at spike i, when: f (i) <10 Hz, or when: f (i+1)<f (i)<20 Hz, where f=spike instantaneous frequency. The threshold for burst detection was set to be lower than the amplitude of LevMN-5 or DepMN-Ds (for levator and depressor nerves, respectively), and greater than that of LevMNs-1-4 or the depressor nerve CIN. Detection was followed by calculating burst CoGs: time points at which the cumulative summed voltage from burst onset is half its total value. Phase relations and burst instantaneous frequencies were calculated based on the CoGs.

### Statistics

Linear data were analysed using STATISTICA 10 software (StatSoft Inc., Tulsa, OK, USA). Data were tested for normality using Shapiro-Wilk test (a=0.05). For statistical analysis of circular data (e.g. phase relations) we used ORIANA 4 software (Kovach Computing Services, Wales, UK). For circular data, normality was defined as a von-Mises distribution and was tested using a single-sample Watson's U^2^ test. Means of von-Mises distributed data were compared using the Watson-Williams F-test, and uniform scores test for other distributions (Zar, 1999).

Correlation coefficients were compared using the Fisher r-to-z transformation (Fisher, 1915), and comparing the resultant z-scores [Cohen and Cohen (1983); formula 2.8.5]. Burst frequency was calculated as instantaneous frequency, and is used here as a representation of stride-frequency in a walking animal, similarly to the cycle-period used as a measure for stride-period. In cases where only one *P*-value is given for a set of comparisons, that *P*-value is the highest among all results of these comparisons.
